# Genetic Risk for Smoking: Disentangling Interplay Between Genes and Socioeconomic Status

**DOI:** 10.1007/s10519-021-10094-4

**Published:** 2021-12-02

**Authors:** Joëlle A. Pasman, Perline A. Demange, Sinan Guloksuz, A. H. M. Willemsen, Abdel Abdellaoui, Margreet ten Have, Jouke-Jan Hottenga, Dorret I. Boomsma, Eco de Geus, Meike Bartels, Ron de Graaf, Karin J. H. Verweij, Dirk J. Smit, Michel Nivard, Jacqueline M. Vink

**Affiliations:** 1 Behavioural Science Institute, Radboud University Nijmegen, Nijmegen, The Netherlands; 2 Department of Medical Epidemiology and Biostatistics, Karolinska Institute, PO Box 281, 171 77 Stockholm, Sweden; 3 Department of Biological Psychology, Vrije Universiteit Amsterdam, Amsterdam, The Netherlands; 4 Amsterdam Public Health Research Institute, Amsterdam University Medical Centers, Amsterdam, The Netherlands; 5 Research Institute LEARN!, Vrije Universiteit Amsterdam, Amsterdam, The Netherlands; 6 Department of Psychiatry and Neuropsychology, School for Mental Health and Neuroscience, Maastricht University Medical Center, Maastricht, The Netherlands; 7 Department of Psychiatry, Yale University School of Medicine, New Haven, CT USA; 8 Department of Psychiatry, Amsterdam UMC, Amsterdam Neuroscience, University of Amsterdam, Amsterdam, The Netherlands; 9 Trimbos-Instituut, Netherlands Institute of Mental Health and Addiction, Utrecht, The Netherlands

**Keywords:** Gene–environment interaction, Gene–environment correlation, Smoking, Wellbeing, Mental health, GWAS, GWAS-by-subtraction, Educational attainment, Socioeconomic status, Neighborhood

## Abstract

This study aims to disentangle the contribution of genetic liability, educational attainment (EA), and their overlap and interaction in lifetime smoking. We conducted genome-wide association studies (GWASs) in *UK Biobank* (N = 394,718) to (i) capture variants for lifetime smoking, (ii) variants for EA, and (iii) variants that contribute to lifetime smoking independently from EA (‘smoking-without-EA’). Based on the GWASs, three polygenic scores (PGSs) were created for individuals from the Netherlands Twin Register (*NTR*, N = 17,805) and the Netherlands Mental Health Survey and Incidence Study-2 (*NEMESIS-2*, N = 3090). We tested gene–environment (G × E) interactions between each PGS, neighborhood socioeconomic status (SES) and EA on lifetime smoking. To assess if the PGS effects were specific to smoking or had broader implications, we repeated the analyses with measures of mental health. After subtracting EA effects from the smoking GWAS, the SNP-based heritability decreased from 9.2 to 7.2%. The genetic correlation between smoking and SES characteristics was reduced, whereas overlap with smoking traits was less affected by subtracting EA. The PGSs for smoking, EA, and smoking-without-EA all predicted smoking. For mental health, only the PGS for EA was a reliable predictor. There were suggestions for G × E for some relationships, but there were no clear patterns per PGS type. This study showed that the genetic architecture of smoking has an EA component in addition to other, possibly more direct components. PGSs based on EA and smoking-without-EA had distinct predictive profiles. This study shows how disentangling different models of genetic liability and interplay can contribute to our understanding of the etiology of smoking.

**Supplementary Information:**

The online version contains supplementary material available at 10.1007/s10519-021-10094-4.

## Introduction

Despite well-known health risks and a worldwide increase of discouragement policies, large proportions of the world’s population continue to smoke (World Health Organization 2019). In the Netherlands, the promising decline in smoking seen in the past decades now seems to level off, especially among young adults (Bommelé and Willemsen 2020). Research into the etiology of smoking could shed new light on possible avenues for prevention and intervention. Both environmental and genetic factors play a role in smoking behavior (Sullivan and Kendler 1999).

Characteristics related to socioeconomic status (SES), with educational attainment (EA) as its core component, are important predictors for smoking (Hiscock et al. 2012). Individuals with lower SES (income and EA) are more likely to get exposed to tobacco smoke, start smoking in adolescence, smoke more heavily, and continue smoking. Such effects can be observed at the level of neighborhoods, with people living in more disadvantaged areas being more likely to smoke (Cambron et al. 2018; Karriker-Jaffe 2013). Reported effects are quite large for specific groups. For example, men have been reported to be two times more likely to smoke in a neighborhood marked by visible signs of disorder (e.g., vandalism and litter) than in a neighborhood low on these signs (Miles 2006). White residents of poor neighborhoods are 72% more likely to initiate smoking before age 25 than white residents in an affluent neighborhood (even after controlling for income and parental education; Kravitz-Wirtz 2016). However, estimated effect sizes vary widely and seem to be moderated by many individual-level SES and group attributes (Cohen et al. 2011; Karriker-Jaffe et al. 2016; Kravitz-Wirtz 2016; Mathur et al. 2013; Miles 2006).

Twin studies estimated that almost half of the individual differences in the population in smoking initiation can be attributed to genetic factors. The heritability estimate is even higher (around 75%) for nicotine dependence (Vink et al. 2005). As the prevalence of smoking seems to be declining and it has become less socially acceptable, heritability estimates have become somewhat higher (Boardman et al. 2010; Vink and Boomsma 2011; Wedow et al. 2018). Genome-wide association studies (GWASs) have identified specific genetic variants underlying smoking behavior (The Tobacco and Genetics Consortium 2010). The most recent smoking GWAS included more than a million participants, and all measured genetic variants could explain 8% of the variation in smoking initiation and 8% in the number of cigarettes smoked per day (Liu et al. 2019). Thus, part of the heritability as estimated by twin studies could not be traced back to common variation tested in this GWAS. There are several possible reasons for this commonly observed ‘missing heritability’, one of which might be interplay with environmental circumstances (Eichler et al. 2010).

### Mixed Findings on Gene–Environment Interaction in Smoking

It seems likely that socioeconomic and genetic factors do not operate in isolation in increasing risk for smoking. In the case of gene–environment interaction (G × E), the likelihood that genetic risk (G) for smoking leads to smoking depends on environmental circumstances (E). Such G × E effects could contribute to the missing heritability phenomenon in two ways (Manolio et al. 2009). First, G × E could contribute to inflated heritability estimates in twin research (Verhulst and Hatemi 2013), meaning that the gap between twin and SNP-heritability is actually smaller than it seems. Second, G × E could deflate associations if the total effect of a SNP is canceled out due to different effects in different subgroups, meaning that it ‘hides’ part of the SNP heritability (Manolio et al. 2009). Thus, testing G × E could help us gain a fuller understanding of the genetic etiology of traits.

Twin studies have suggested that G × E effects exist for smoking (e.g., Boardman et al. 2011, 2008; Dick et al. 2007; Timberlake et al. 2006). For example, EA was found to moderate the heritability of smoking initiation (although the exact direction was difficult to establish due to strong gene–environment correlation effects; McCaffery et al. 2008). However, such studies do not provide any insight as to what genetic variants drive these G × E effects. More recently, studies have used smoking GWASs to create polygenic scores (PGSs) as a measure of genetic risk, and tested interaction between PGSs and environmental factors on smoking. For example, it was shown that a PGS for smoking initiation was associated with smoking heaviness only in individuals who had been exposed to tobacco smoke in childhood (Treur et al. 2018). Another study showed that a smoking PGS was more likely to contribute to smoking risk in individuals that had experienced trauma than in individuals who had not (Meyers et al. 2013). Similarly, it was found that a PGS for smoking predicted smoking more strongly in sample of war veterans than in non-veterans (Schmitz and Conley 2016). On the other hand, living in a neighborhood with high social cohesion buffered for genetic risk, such that the effect of the PGS on smoking was less strong for individuals living in such neighborhoods (Meyers et al. 2013). However, a recent study did not detect G × E with neighborhood-level SES and metropolitanism on smoking (Pasman et al. 2020). Overall, the evidence for G × E in PGS studies is somewhat mixed for smoking (Pasman et al. 2019). Also, given the small effect sizes of PGSs in general and the even smaller G × E effects, these studies have done little to solve the missing heritability.

### Gene–Environment Correlation and Other Types of G–E Interplay in Smoking

G × E research has often been framed in terms of environmental exposures that moderate genetic risk factors. However, the distinction between ‘environmental’ exposures and other characteristics is often quite difficult to make. For instance, an interaction with sex could indicate biological differences in the chance that some genetic factor will come to expression or could indicate an environmental effect of gender roles. Moreover, many factors that are thought of as environmental (e.g., the parenting and social environment, Vinkhuyzen et al. 2010) are actually heritable themselves, so that the environment and the genetic make-up become associated. This phenomenon is often referred to as gene–environment correlation (rGE). There are various mechanisms by which associations between an environmental exposure and genetic predisposition can arise. For example, given that parents and offspring share part of their genetic make-up, a correlation could arise between parenting behavior and offspring genes (passive rGE, Kong et al. 2018; Pasman et al. 2021; Plomin et al. 1977). Alternatively, a correlation between an individual’s risk for smoking and the environment could arise because smoking elicits some response in other people (reactive rGE) or because smokers select different environments for themselves (active rGE; Plomin et al. 1977). Such rGE effects may also exist for EA, which has a substantial genetic component. Both cognitive abilities (at the core of EA) as well as non-cognitive EA-traits and socioeconomic characteristics have been shown to be heritable traits (Demange et al. 2020; Marioni et al. 2014). Given the strong association between EA and smoking, down the line rGE associations could arise between genetic risk for smoking and EA.

Such rGE effects influence the interpretation of other genetic findings. First, rGE effects (with shared environment) could inflate heritability estimates in twin research when not explicitly modeled (Verhulst and Hatemi 2013). Second, they can lead to the detection of environmental signal in GWASs (Manolio et al. 2009; Shen and Feldman 2020). For example, GWASs will probably pick up on different variants for smoking in an environment that highly sanctions smoking (e.g., variants associated with risk taking and addiction-proneness) than in an environment where smoking is the norm (e.g., variants associated with social behavior), giving rise to rGE between smoking variants and social norms. Third, if there are rGE effects, this can change the interpretation of G × E effects, lower the chance that G × E will be detected, or lead to spurious G × E findings (Dudbridge and Fletcher 2014; Rathouz et al. 2008).

The plausibility that rGE exists in substance use has been widely acknowledged (Gage et al. 2016; Kong et al. 2018) and there are indications for the existence of rGE in the smoking literature. Some twin studies have shown that peer behavior is associated with genetic risk for smoking in adolescents (Cleveland et al. 2005; Harden et al. 2008; Wills and Carey 2013). This has commonly been interpreted as showing that genetic risk for smoking somehow influences which friends adolescents select for themselves. One study using PGS to test rGE showed overlap between the parenting environment and a smoking PGS (Pasman accepted). Another study showed rGE between a smoking PGS and neighborhood ‘physical disorder’ (i.e., disrepair and vacancy; Meyers et al. 2013). A last study went a step further and showed not only strong rGE between genetic risk for smoking and EA, but showed that this rGE relationship depended on birth cohort (Wedow et al. 2018). rGE was stronger in younger cohorts, where smoking had become rarer and was more strongly influenced by genetic factors, than in older cohorts, where smoking was a more common social phenomenon. This moderated rGE effect implies that genetic relationships can be mediated through environmental exposures and adds a layer of complexity to thinking about gene–environment interplay. Together, these findings suggest that genetic associations from smoking genetics studies must be interpreted strictly within the environmental context of the samples. Still, studies reporting rGE, especially those using PGS, are scarce.

### Partitioning Genetic Vulnerability to Smoking in EA and Non-EA Components

The first aim of this study is to disentangle genetic effects that influence smoking through (rGE with) EA from other, more direct genetic components. That is to say, we model the genetic predisposition for EA and subtract it from the total genetic liability for smoking. This way, we can assess the contribution of EA (with its rGE components) in the etiology of smoking, and compare it to a ‘cleaner’ genetic component of smoking effects that are independent from EA. Second, we aim to test if the PGS based on either these more direct smoking variants (PGS_smok-noEA_) or the EA variants (PGS_EA_) pick up better on G × E effects, as compared to a general PGS based on all smoking variants taken together (PGS_allsmok_). We test interactions with neighborhood quality and affluence. The G × E effects per PGS could go in different directions. On the one hand, if it is true that rGE effects dilute G × E effects (and we have taken some of them out of the equation by regressing out EA), the PGS assessing the more direct smoking effects could be more sensitive for picking up G × E. In this case, individuals with a high PGS_smok-noEA_ may react more strongly to an unfavorable neighborhood environment and have a higher chance to start smoking. On the other hand, it is also possible that individuals who are genetically liable for a high-risk environment react differently to that environment than people who are not. That is to say, individuals with a high PGS_EA_ may be vulnerable to the environment, whereas people with a high genetic risk for smoking (high PGS_smok-noEA_) have a higher chance to start smoking regardless of the environment. Comparing G × E effects between PGSs for all-smoking, smoking-without-EA, and EA could contribute to formulating such competing hypotheses and shed more light on interplay between genetic and environmental vulnerability for smoking.

## Methods

First, GWA analyses were performed to estimate SNP effects on smoking and EA. Second, using the results from these GWASs, EA effects were subtracted from smoking effects to capture smoking-without-EA. Third, polygenic scores were created to conduct follow-up tests of G × E effects with measures of EA and neighborhood SES. The first two steps were conducted using data from the *UK Biobank*, the third step was conducted in two independent samples from the Netherlands Mental Health Survey and Incidence Study-2 (*NEMESIS-2*) and the Netherlands Twin Register (*NTR*).

### Samples and Measures: UK Biobank

The GWA analyses on smoking, EA, and smoking-without-EA were conducted in a sample from the *UK Biobank*. The *UK Biobank* contains phenotypic and genetic information from up to 500,000 inhabitants of the United Kingdom. It has received ethical approval from the National Health Service North West Center for Research Ethics Committee (reference: 11/NW/0382). Researchers can apply for access to this rich data set to conduct health-related studies. This study was conducted under project number 40310. For our analyses we selected N = 394,718 individuals from European ancestry for whom there was complete phenotypic and genotypic information. Mean age was M = 56.8 (range 39–73, SD = 8.0) and 54.2% of the sample was female.

To measure lifetime smoking in the *UK Biobank* we extracted information from all measurement instances of data fields 2867 and 2897 (age at smoking initiation), 2887 and 3456 (cigarettes per day), and 20,116 (smoking initiation yes/no). People indicating on field 2887 or 3456 to (have) smoke(d) one or more cigarettes per day were classified as smokers. People indicating on field 20,116 to never have been a smoker were classified as non-smokers. If field 2887 and 3456 were unavailable, but people indicated on field 20,116, 2867, or 2897 to be an (ex-) smoker, they were classified as smokers. There were data for 272,943 (54.60%) never smokers and 226,795 lifetime smokers. To capture EA, we used the ISCED classification to transform reported educational levels from field 6138 to a standardized number of educational years (UNESCO Institute for Statistics 2011). We selected the highest reported completed educational level and classified ‘none of the above’ (N = 90,360) as primary school only. Average years of education was M = 14.93, SD = 5.12, range = 7–20, N = 451,800.

### Samples and Measures: *NTR*

In the second part of the study, we use data from two independent samples from the Netherlands. The Netherlands Twin Register (*NTR*) is an ongoing longitudinal study of twins and their families which has been described in detail elsewhere (refer to Ligthart et al. 2019, also for a description of the genetic data). We included all available measures of smoking initiation that were collected between 1991 and 2019 (from 15 different surveys, see Supplementary Table S1). Part of the sample is followed longitudinally, and new participants have been recruited continuously. In order to maximize sample size, we selected the most recent available measurement of smoking status for all participants. For N = 14,618 European ancestry adult individuals, there were genome-wide SNP and complete phenotypic data. For most participants, smoking data were collected between 2013–2016 (N = 9426) or in 2009 (N = 1361; see Table S1). At the time of phenotype measurement, mean age was M = 43.31 (SD = 17.12, range = 18–94). Lifetime smoking was defined similarly as in the *UK Biobank*. Current and ex-smokers that (previously) smoked more than occasionally (1 cigarette per day or 7 per week) were classified as smokers. Occasional and never smokers were classified as non-smokers. The sample consisted of 63.1% females and 43.1% lifetime smokers.

To measure neighborhood SES (E in the G × E analysis) we focused on the average household income in the neighborhood of residence. We identified the first four digits of the postal code of the participant at the time of measurement of the smoking phenotype, corresponding to the residential area at the level of neighborhoods. These digits were coupled to governmental registration data on neighborhood-level income (Centraal Bureau voor de Statistiek (CBS) 2012). The CBS determined average monthly income per household before tax (rounded at hundreds) in 2004 and 2010. We used the neighborhood data that were closest in time to survey used to assess lifetime smoking. Data were available for N = 12,584 participants, who on average lived in neighborhoods with a per-household monthly income of M = 2678.64 (SD = 934.40, winsorized at min = 500 and max = 10,000).

In the follow-up analyses we focused on satisfaction with life, which was available in 4 different surveys. It was measured using the translated Satisfaction with Life Scale (Arrindell et al. 1999; Diener et al. 1985), a survey with five 7-point Likert items on how happy people are with their life. The sum score on this scale was coupled to contemporaneous neighborhood income using similar procedures as before, prioritizing the measurements closest in time to the measure of neighborhood income. Average satisfaction with life was M = 26.97 (SD = 5.23, range = 5–35, N = 9257).

### Samples and Measures: *NEMESIS-2*

The Netherlands Mental Health Survey and Incidence (*NEMESIS-2*) is a population sample of more than 6500 individuals that were followed in four measurement waves spaced out between 2007 and 2018. The aim was to monitor the occurrence and course of common mental disorders in the general population (De Graaf et al. 2010). For this study, we used data from the second wave (conducted in 2010–2012), where a measure of neighborhood quality was available. For a sub sample of N = 3090 European-ancestry individuals genetic and phenotypic data were available. About half of the sample was female (56.1%), and mean age at wave 2 was 47.2 (SD = 12.5, range = 21–71).

To assess smoking we used questionnaire items on smoking status. People were classified as smokers if they self-identified as current or ex-smokers; (former) occasional smokers were classified as never-smokers. A third of the sample classified as lifetime smokers (30.2%). To measure neighborhood quality, we used a sum score of 5 standardized Likert scale survey items, including appreciation of the neighborhood, frequency of noise from neighbors, traffic, or other sources in the neighborhood, frequency of feeling unsafe if walking alone in the neighborhood during the day, frequency of feeling unsafe if walking alone in the neighborhood during the night, and frequency of observing vandalism. Items were re-coded in the positive direction, such that a higher score means a higher neighborhood quality.

Since neighborhood quality was not measured at baseline, we used wave 2 data. There was some attrition from baseline (N = 319), which incited us to employ the automatic multiple imputation procedure from SPSS to supplement wave 2 neighborhood quality. We used 32 unique sociodemographic measures as predictors (see Supplementary Table S2). Because each imputed value is subject to some random variation, we imputed 25 datasets and interpret the pooled results. In total, 10.3% of the neighborhood quality data were imputed using this procedure. Across analyses, we compared the pooled results with the results using the original data, and saw that differences were negligible. For the smoking outcome, we carried forward baseline data in case they were missing at wave 2. In follow-up analysis we looked at mental health as outcome. To measure this, we used a clinical rating if someone had met criteria for any DSM-IV axis-I disorder since the baseline measurement. If wave 2 data were unavailable while someone had met criteria for a disorder at baseline, we carried forward the baseline data (N = 92 individuals). Remaining missingness (N = 227) was imputed using the same baseline predictors as before. DSM-IV contains 18 disorder categories (including for example mood and psychotic disorders) with in total almost 300 different diagnoses. Diagnoses were made based on the Composite International Diagnostic Interview (CIDI) 3.0 by a trained professional (De Graaf et al. 2010). In total, 541 of the participants (17.5%) had recently met criteria for any disorder at wave 2 (since the last interview or in the past year for individuals who only had wave 1 data).

### GWAS in UK Biobank to Model EA and Non-EA Effects on Smoking

**Fig. 1:**
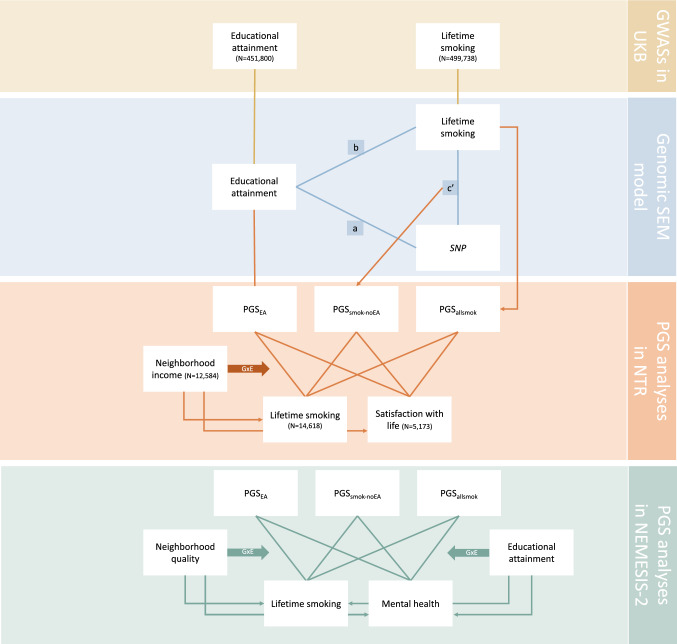
Flow-chart of the different analysis phases, with each phase in a different colour. In the Genomic SEM model, path (a) denotes the EA GWAS (direct SNP-EA associations), path (b) the smoking GWAS (direct SNP-smoking associations), and path (c’) the SNP-smoking association remaining after controlling for mediation through EA, capturing SNP effects on smoking-without-EA. In the *NEMESIS-2* sample we used multiple imputation, such that N was constant (N = 3090) for all analyses. *PGS* PolyGenic Score, *EA* educational attainment, *PGS*_*EA*_  PGS for EA, *PGS*_*smok-noEA*_  PGS for smoking-without-EA, *PGS*_*allsmk*_  PGS for lifetime smoking (Color figure online)

In the first step, we ran GWASs to capture genetic associations for lifetime smoking and EA (yellow panel in Fig. 1). We used the fast-GWA package from GCTA (Yang et al. 2011). GCTA makes use of a genetic relatedness matrix to account for relatedness in the sample. To reduce computational demand for subsequent analyses we limited the GWASs to 1.3 million HapMap3 SNPs (International HapMap 3 Consortium 2010). We filtered out SNPs with minor allele frequency below 1%, divergence from Hardy–Weinberg disequilibrium with *p*_*HWE*_ < 10^–10^, and call rate below 95%. We included genetic sex, standardized age, standardized year of birth, and 25 principal components (PCs) for genetic ancestry as covariates. These PCs were determined using PCA, as described in more detail in Abdellaoui et al. (2019).

In the next step, we used the summary statistics to fit a mediation model capturing genetic effects on smoking, EA, and smoking-without-EA in Genomic Structural Equation Modeling (Genomic SEM, Grotzinger et al. 2018). To obtain a smoking-without-EA GWAS, we regressed smoking on all genetic variants as well as on EA (blue panel, Fig. 1). The model yielded two sets of GWAS results, one for SNP effects on smoking independent from EA (‘smoking-without-EA’, grey path) and one for SNP effects on EA (red path from SNP to EA). We inspected the GWAS results and performed post-processing analyses using FUMA on default settings to inspect the genetic architecture of the different traits (version v1.3.6a, Watanabe et al. 2017). We used LDscore regression (Bulik-Sullivan et al. 2015) to assess SNP-based heritability (the variance explained in the traits by all SNPs concurrently) and genetic correlations with other traits. SNPs and genes that were genome-wide significantly associated with one of our traits were looked up in the GWAS catalog from EMB-EBI (Buniello et al. 2019) to examine whether they were previously associated with other phenotypes.

### Polygenic Score Analysis to Test Genetic and SES Influences on Smoking

PGS were created in *NTR* and *NEMESIS-2* based on the total smoking GWAS, EA, and smoking-without-EA summary statistics from the Genomic SEM model. A PGS can be created in a new sample by weighting variants by their GWAS effect size and aggregating them in a single score per individual. We used GCTA-SBLUP to take into account the linkage disequilibrium (LD) structure in the European population before creating the PGS, as this improves prediction accuracy (Robinson et al. 2017; Yang et al. 2011). An additional advantage of SBLUP is that no *p*-value threshold needs to be established for including SNPs in the PGS (as is the case for some other PGS computation methods); rather, the whole genome is weighted and integrated in the score. Using the SBLUP weighting scheme, the actual individual-level scores were computed with PLINK (Purcell et al. 2007) and merged to the phenotypical data in SPSS.

We tested the associations between these PGSs and lifetime smoking in *NTR* and *NEMESIS-2*. In order to compare the different PGS components, we first regressed the smoking outcome on the ‘all smoking’ PGS (PGS_allsmok_; *model 1a*), and then on the PGS for EA (PGS_EA_) and the PGS for smoking-without-EA (PGS_smok-noEA_) together to assess their relative contribution (*model 1b*). All continuous variables were standardized. Covariates included in the model were age, sex, and the first ten principal components for genetic ancestry (PCs). In addition, in *NTR* we controlled for the genotyping batch, as several different SNP arrays have been used over the course of data collection. Also, because of the family structure in *NTR,* we used generalized estimating equations (GEE) to correct for clustering in this sample, whereas standard logistic regression could be employed in *NEMESIS-2*. Secondly, we included a measure of neighborhood quality to test its effect on smoking in the two different PGS models (*model 2a and 2b*). Third, we added neighborhood quality x PGS terms, comparing a model with the PGS_allsmok_ (*model 3a*) with a model with the PGS_SES_ and the PGS_smok-noSES_ (*model 3b*). In models 3a-b, we added the interaction terms between the PCs, the PGSs, and the neighborhood predictor (Keller 2014). Finally, we repeated these analyses with a measure of satisfaction with life (in *NTR*) and mental health (in *NEMESIS-2*) to see if the effects of PGS_EA_ and PGS_smok-noEA_ are specific to smoking, or have a wider impact. If the PGS_smok-noEA_ shows no relationship to mental health, this would be in support of our effort to ‘regress out’ EA effects, indicating that it captures genetic effects specific to smoking. To correct for multiple testing, we divided a conventional 0.05 *p*-value threshold by eight independent tests (2 samples, 2 outcomes, 1 group of interdependent genetic predictors, and 2 neighborhood predictors) resulting in a threshold of *p* < 0.006. To compute R^2^ of the individual PGSs, we regressed the outcomes on the PGS and the genetic covariates (genotyping batch and PCs; the genetic covariates hardly added any explained variance, data not shown). As R^2^ is not provided in GEE analyses, we were unable to control for family structure here.

## Results

The results of the GWASs for smoking and EA in *UK Biobank* can be found in the supplement. Supplementary Tables S3-4 show the independent genome-wide significant risk loci for the traits (at R^2^ < 0.1 and distance > 250 kb). In Supplementary Figures S1-2 Manhattan plots are presented. There were 112 independent variants identified for lifetime smoking, with the strongest association with a SNP in *NCAM1* on chromosome 11*.* SNP-based heritability (h^2^) for lifetime smoking was 9.2% (SE = 0.29). The GWAS of EA identified 276 independent significant loci (h^2^ = 14.2%, SE = 0.42), with the strongest SNP rs9372625 in *AL589740.1* on chromosome 6. The SNP h^2^ for EA was 14.2%.

### Modeling Direct Genetic Effects and EA Effects on Smoking

Using Genomic SEM, we fitted the model represented in Fig. 1 (blue panel) based on the genetic correlations between traits as measured by the summary statistics from the conducted GWASs. We tested a mediation model with the SNPs as the predictors, smoking as the outcome, and EA as the mediator. We were interested in path c’, representing the genetic effects on smoking that remained after taking into account the effects that were mediated by EA. The summary statistics for c’ thus constitute smoking-without-EA.

**Fig. 2:**
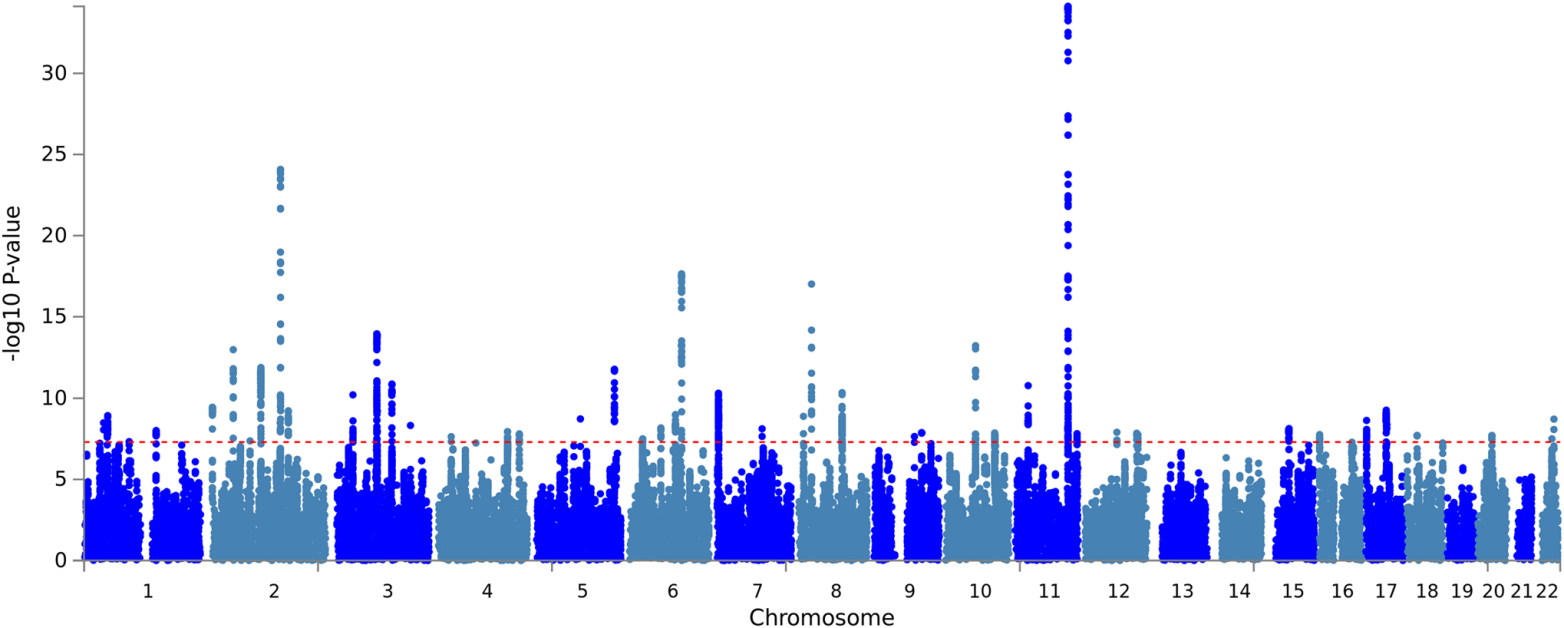
Manhattan plot for the GWAS on smoking-without-EA, where EA effects were subtracted from the smoking GWAS in Genomic SEM. The red line denotes the genome-wide significance threshold of *p* = 5E−08 (Color figure online)

The GWAS for smoking-without-EA identified 47 genetic loci (Table S5 and Fig. 2) and yielded a SNP-based heritability of 7.2% (SE = 0.28). The top SNP was rs10891487, an intron variant in the *NCAM1* gene. This SNP and its LD partners have been associated with traits related to risk taking, substance use, cognitive ability, and socioeconomic status (Table S6). The strongest associations on the gene-level were found for *NCAM1* on chromosome 11 and *CADM2* on chromosome 3 (Table S7; Figure S3). *NCAM1* was already a top-gene for smoking before controlling for shared effects on EA (Figure S1), whereas the effect of *CADM2* was boosted after controlling for EA. Both genes have been implicated in numerous risk and substance use behaviors (Table S6), are highly brain expressed, and play a role in neuronal cell adhesion.

**Fig. 3:**
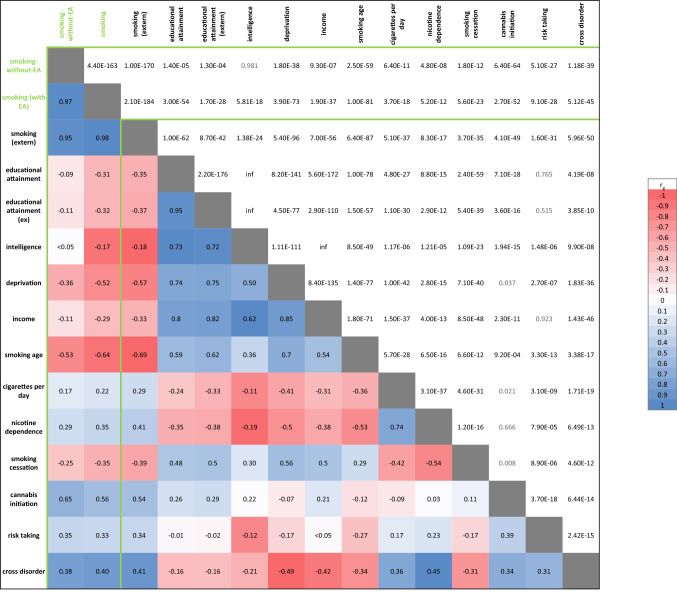
Heat map of genetic correlations between the smoking-without-EA GWAS and SES- and smoking related traits. Below the diagonal are the correlation estimates, with colors indicating the direction (red = negative; blue = positive) and strength (dark = strong; light = weak) of the association. Above the diagonal corresponding p-values are reported, with in grey those that were not significant after correcting for multiple testing with 15 traits (*p* = 0.05/15 = 0.002). Trait description and sources can be found in Supplementary Table S8 (note: deprivation and income summary statistics were derived from our own GWAS analysis of the UK-Biobank sample) (*EA* educational attainment, *extern* same trait but from independent GWAS source, *inf* infinitesimal) (Color figure online)

We performed sensitivity analyses to check if the Genomic SEM model succeeded in capturing smoking-without-EA by computing genetic correlations between smoking-without-EA and other traits (Table S8). Results are summarized in Fig. 3. The genetic correlation between smoking-without-EA and the original smoking trait was r_g_ = 0.97, suggesting that the genetic architecture of smoking was only mildly affected by subtracting EA effects. The correlations with EA (*UK Biobank* summary statistics as well as GWAS summary statistics from an external, independent sample; see Supplementary Table S8 for the source of the used summary statistics) were greatly reduced as compared to the original association (original: r_g_ =  − 0.35; after subtraction: r_g_ =  − 0.09), suggesting that we largely succeeded at subtracting EA effects. Genetic correlations between smoking-without-EA with other SES-indicators (neighborhood deprivation and income) were similarly attenuated. The correlation between smoking and intelligence disappeared after subtracting EA, showing that both cognitive and SES-related components of EA were partialled out. The correlations with smoking-related traits (age at initiation, cigarettes per day, nicotine dependence, cessation, cannabis initiation, and risk-taking behavior) were also attenuated, but less so; this attenuation likely represents some signal loss resulting from the subtraction. The correlation between smoking and psychopathology (‘cross disorder,’ the genetic vulnerability across different disorders) remains virtually unchanged after subtracting EA, suggesting that this association is not (completely) driven by overlap of psychopathology and smoking with EA.

### Polygenic Scores

Table 1 presents the results of the PGS analyses in *NTR* and *NEMESIS-2*, showing the association of the PGSs based on the EA and smoking-without-EA GWAS with lifetime smoking (parameter estimates for the full results including genetic covariates can be found in Tables S9a and S10a). In all models, all PGSs significantly predicted lifetime smoking. Individually, the PGS_allsmok_, PGS_EA_, and PGS_smok-noEA_ explained respectively 3.1%, 2.2%, and 0.5% of the variance in smoking in *NTR*, and 4.4%, 2.3%, and 0.8% in *NEMESIS-2*. Combined into the same model, the PGSs explained at total of 6.3% of the variance in smoking in *NTR* and 4.5% in *NEMESIS-2*. The effect of PGS_EA_ on smoking was negative, such that having a genetic predisposition for a higher EA was associated with lower chances of being a smoker.

**Table 1:** Results of the polygenic score (PGS) analyses in the *NTR* and *NEMESIS-2* sample with *lifetime smoking* as outcome

	Lifetime smoking *NTR* (N = 12,584–14,618)^a^				Lifetime smoking *NEMESIS-2* (N = 3090)			
	b	SE	OR	*p*	b	SE	OR	*p*
0								
Age	0.706	0.021	2.03	**1.46E − 252**	** − **0.125	0.083	0.883	0.133
Sex^b^	** − **0.291	0.036	0.748	**9.46E − 16**	** − **0.158	0.042	0.854	**2.07E − 4**
	R^2^ = 11.7%				R^2^ = 1.9%			
1a								
PGS_allsmok_	0.389	0.019	1.476	** < 1E − 320**	0.354	0.042	1.424	** < 1E − 320**
Age	0.652	0.022	1.919	** < 1E − 320**	− 0.165	0.043	0.848	**1.38E − 04**
Sex	− 0.273	0.037	1.314	**1.41E − 13**	− 0.141	0.084	0.869	0.095
	R^2^ = 16.3% (Δ = 4.6%)				R^2^ = 5.4% (Δ = 3.5%)			
1b								
PGS_EA_	− 0.223	0.020	0.800	** < 1E − 320**	− 0.462	0.059	0.630	**5.33E − 15**
PGS_smok-noEA_	0.347	0.019	1.415	** < 1E − 320**	0.335	0.058	1.398	**8.36E − 09**
Age	0.663	0.022	1.941	** < 1E − 320**	− 0.169	0.043	0.845	**9.49E − 05**
Sex	− 0.272	0.037	1.313	**1.84E − 13**	** − **0.138	0.084	0.871	0.100
	R^2^ = 16.8% (Δ = 5.1%)				R^2^ = 4.9% (Δ = 3.0%)			
2a								
PGS_allsmok_	0.396	0.022	1.486	** < 1E − 320**	0.354	0.042	1.425	** < 1E − 320**
Neighborhood	** − **0.158	0.023	0.854	**1.77E − 11**	0.071	0.042	1.073	0.091
Age	0.798	0.028	2.222	** < 1E − 320**	− 0.167	0.043	0.846	**1.19E − 04**
Sex	** − **0.273	0.043	1.314	**1.89E − 10**	** − **0.148	0.084	0.863	0.080
	R^2^ = 18.2% (Δ = 6.5%)				R^2^ = 5.6% (Δ = 3.7%)			
2b								
PGS_EA_	** − **0.196	0.022	0.822	** < 1E − 320**	** − **0.461	0.059	0.631	**6.44E − 15**
PGS_smok-noEA_	0.359	0.022	1.432	** < 1E − 320**	0.336	0.058	1.399	**7.91E − 9**
Neighborhood	** − **0.145	0.023	0.865	**5.32E − 10**	0.064	0.042	1.067	0.123
Age	0.804	0.028	2.235	** < 1E − 320**	** − **0.171	0.043	0.843	**8.40E − 05**
Sex	** − **0.272	0.043	1.312	**2.56E − 10**	** − **0.145	0.084	0.865	0.086
	R^2^ = 18.4% (Δ = 6.7%)				R^2^ = 5.2% (Δ = 3.3%)			
3a								
PGS_allsmok_	0.398	0.022	1.489	** < 1E320**	0.361	0.043	1.435	** < 1E320**
Neighborhood	** − **0.171	0.024	0.843	** < 1E320**	0.027	0.054	1.028	0.616
PGS_allsmok_ × neigh	** − **0.046	0.022	0.955	0.034	** − **0.028	0.050	0.972	0.571
Age	0.801	0.028	2.228	** < 1E320**	** − **0.171	0.044	0.843	** < 1E320**
Sex	0.278	0.043	1.320	** < 1E320**	** − **0.135	0.087	0.874	0.122
	R^2^ = 18.5% (Δ = 6.8%)				R^2^ = 6.5% (Δ = 4.6%)			
3b								
PGS_EA_	** − **0.190	0.023	0.827	** < 1E320**	** − **0.257	0.044	0.774	** < 1E320**
PGS_smok-noEA_	0.360	0.022	1.433	** < 1E320**	0.289	0.043	1.335	** < 1E320**
Neighborhood	** − **0.163	0.024	0.850	** < 1E320**	0.037	0.056	1.038	0.504
PGS_EA_ × neigh	0.053	0.022	1.054	0.014	0.009	0.049	1.009	0.852
PGS_smok-noEA_ × neigh	** − **0.031	0.021	0.969	0.141	** − **0.024	0.050	0.976	0.629
Age	0.809	0.028	2.246	** < 1E320**	** − **0.174	0.044	0.840	** < 1E320**
Sex	0.277	0.043	1.319	** < 1E320**	** − **0.145	0.088	0.865	0.098
	R^2^ = 18.9% (Δ = 7.2%)				R^2^ = 8.1% (Δ = 6.2%)			

Models include the effects of the PGS based on EA and the PGS based on smoking-without-EA, main effects of neighborhood environment (income in *NTR*; quality in *NEMESIS-2*), and interaction between PGSs and neighborhood. Covariates in all models 0-3b included age, sex, and the first 10 principal components (PCs) for genetic ancestry; in models 3a-b we also added the interaction terms between the PGSs, PCs, and neighborhood (parameters estimates for all predictors can be found in Supplementary Tables S9a and S10a). Effects with *p* < 0.006 (corrected for 8 independent tests) are bold-faced. Explained variance (R^2^ of the total model is given, with the difference to the null model (Δ)

*PGS* polygenic score, *allsmok* all smoking, *EA* educational attainment, *smok-noEA* effects on smoking independent from EA, *Neighborhood (neigh)* neighborhood characteristics, in NTR neighborhood-level income, in NEMESIS-2 neighborhood quality

^a^Due to missingness in the neighborhood measure, model 2 and 3 had a sample size of N = 12,584

^b^Sex was coded 1 = male, 2 = female

In *NTR*, higher neighborhood income was associated with lower chances of smoking (R^2^ = 1.9% for neighborhood only). There were no significant G × E interactions after correction for multiple testing, although the interactions did add a small amount of explained variance (about 0.2%; neighborhood-by- PGS_allsmok_ *p* = 0.034, neighborhood-by-PGS_EA_ *p* = 0.014, neighborhood-by-PGS_smok-EA_ *p* = 0.141). In all cases the directions were such that the effect of the PGS was stronger for people living in a lower income neighborhood. The model with all effects combined (main, interactions and covariates) explained 18.9% of the variance in lifetime smoking. In *NEMESIS-2*, neighborhood quality was not a significant predictor of smoking. There were no interactions between neighborhood quality and any PGS. All effects combined explained 8.1% of the variance in lifetime smoking.

One of the aims of the PGS_smok-noEA_ was to capture genetic variation that was less diluted by rGE with EA. As a crucial first sensitivity analysis to test if this goal was achieved, we regressed EA and neighborhood-SES on the different PGSs (controlling for genetic covariates and sex and age; see Table 2). The PGS_allsmok_ and PGS_EA_ significantly predicted EA in both *NTR* and *NEMESIS-2.* In *NTR,* PGS_allsmok_ and PGS_EA_ also showed rGE with neighborhood income. Crucially, the relationship between PGS_smok-noEA_ and EA was greatly reduced in both samples, and there was no rGE between the PGS_smok-noEA_ and neighborhood-SES. Thus, it seems that we largely succeeded in excluding rGE with neighborhood-SES by subtracting EA effects from the smoking PGS. Even if partialling out EA effects is unlikely to remove all rGE from smoking, our approach was effective for targeting the rGE with neighborhood-SES.

**Table 2:** Relationships between the PGSs and measures of educational attainment and neighborhood-SES, controlled for genetic covariates (10 PCs in both samples as well as genotyping batch in *NTR*) and sex and age

	*NTR* (*N* = 8989 for EA and *N* = 12,584 for neighborhood)			*NEMESIS-2* (*N* = 3090)		
	PGS_allsmok_	PGS_EA_	PGS_smok-noEA_	PGS_allsmok_	PGS_EA_	PGS_smok-noEA_
EA^a^						
b	− 0.059	0.221	− 0.016	− 0.08	0.228	− 0.031
SE	0.009	0.008	0.009	0.015	0.014	0.014
*p*	**6.24E − 12**	**2.12E − 144**	0.070	**4.80E − 08**	**2.28E − 59**	0.030
R^2^*	2.3%	7.7%	1.8%	1.0%	8.1%	0.2%
Neighborhood^b^						
b	− 0.039	0.126	− 0.011	0.027	0.001	0.025
SE	0.010	0.0116	0.0104	0.08	0.019	0.018
*p*	**1.63E − 04**	** < 5E − 300**	0.281	0.147	0.950	0.177
R^2^	0.6%	2.0%	0.5%	0.3%	0.2%	0.3%

The relationships were tested in separate models, so that these models do not control for overlap between the PGSs

^a^Educational attainment. In *NTR*, 4-level variable with 1 = primary school, 2 = lower vocational/ lower secondary school, 3 = intermediate vocational/intermediate and high secondary school, and 4 = higher vocational/ university; in *NEMESIS-2*, 3-level variable with 1 = primary/lower secondary, 2 = higher secondary, 3 = higher professional education

^b^In *NTR*, a measure of neighborhood-level income; in *NEMESIS-2,* a survey-based measure of neighborhood quality

*R^2^ is given for the model excluding age and sex

Because in *NEMESIS-2* there was no main effect of neighborhood quality, it was not to be expected that it would augment PGS effects in a G × E. As a second sensitivity analysis, we therefore repeated the PGS tests with baseline EA (low, medium, high) as an alternative measure of SES (replacing neighborhood quality). We found a significant, negative association between EA and lifetime smoking, but there were no significant G × E effects (*p* = 0.072–0.242). The interactions did explain some variance in smoking (0.7–0.9%; Supplemental Table S11a), and followed a pattern such that PRS_EA_ only had effects on low to medium levels of education, whereas the effect of PRS_smok-noEA_ and PRS_allsmok_ were stronger at higher levels of education.

As a final sensitivity analysis, we repeated the analyses for measures of mental health and wellbeing (see Supplementary Table S9b, S10b and S11b). In *NTR*, higher PGS_EA_ and higher neighborhood income significantly predicted more satisfaction with life (R^2^ = 1.0% and 2.0%, respectively); the effects of PGS_allsmok_ and PGS_smok-noEA_ were not significant (R^2^ = 0.4% and 0.1%). There were patterns for G × E between neighborhood income and PGS_allsmok_ and PGS_smok-EA_, but these did not survive correction for multiple testing. The direction was such that the smoking PGSs had a negative effect on satisfaction with life only at high neighborhood income (both R^2^ < 0.1%, *p* = 0.026 and *p* = 0.025*,* respectively). In *NEMESIS-2*, PGS_EA_ was significantly negatively related to the risk of having a recent diagnosis of a psychiatric disorder (R^2^ = 1.2%), as was neighborhood quality (1.1%). The PGS_allsmok_ predicted mental health less strongly than the PGS_EA_ (only reaching significance in the models including neighborhood quality, R^2^ = 0.6%), and the effect of the PGS_smok-noEA_ on mental health did not reach significance (R^2^ = 0.4%). There were no G × E patterns for mental health in *NEMESIS-2.*

## Discussion

This study showed that the genetic signatures for educational attainment (EA) and smoking overlap substantially, but EA effects can be disentangled to some extent from smoking. After ‘subtracting’ EA effects from the genetic architecture of smoking, still 7.2% of the variance in smoking could be explained by SNP effects (as compared to 9.2% before subtracting). This suggests that the more ‘direct’ component of the genetic variance is important, and not all variance in smoking can be explained through gene–environment correlation (rGE) with EA. We showed that the genetic correlations of smoking with EA and SES-related traits were reduced after subtracting EA, whereas the correlations with smoking traits were less affected. Thus, our approach to subtracting the EA component from the genetic architecture of smoking was successful.

Polygenic scores (PGS) based on the regular smoking GWAS (‘all-smoking’), the EA GWAS, and the GWAS for smoking independent from overlap with EA (‘smoking-without-EA’) all significantly predicted smoking in two independent samples. The PGS for all-smoking explained the largest amount of variance in smoking, followed by the PGS for EA. Thus, the ‘smoking-without-EA’ effects had lower predictive power, in spite of its substantial SNP-heritability and cleaner signal. This lower predictive ability could be simply due to loss of statistical power, or might indicate that genetic predisposition for EA actually contributes more strongly to smoking than comparatively more ‘direct’ genetic smoking effects. This suggestion aligns with research showing that genetic risk factors for smoking initiation are often of a more general behavioral nature, including for example genes associated with risk taking proneness, as compared to risk factors for smoking quantity and nicotine dependence, that are more related to the biological effects of smoking (Karlsson Linnér et al. 2019; Liu et al. 2019; Wang and Li 2010). However, it should be noted that it is likely that we also subtracted some ‘real’ smoking effects in our smoking-without-EA factor. For example, if a variant causes lifetime smoking, and smoking in turn causes lower EA (or vice versa; Gage et al. 2018, 2020), subtracting EA would eliminate the effect of that smoking variant. Such mechanisms may have contributed to the lower genetic signal in the smoking-without-EA GWAS, and the lower predictive power of its PGS.

For mental health and wellbeing we observed a contribution of genetic effects for EA, but no effects of the smoking-without-EA PGS, suggesting that these PGSs indeed captured what was purported. The variance explained by the EA PGS was higher than the variance explained by the all-smoking PGS, which captured both EA and smoking effects, which shows that taking into account genetic smoking effects diluted rather than strengthened the predictive power. This could indicate that previously observed (genetic) associations between smoking and mental health/ wellbeing outcomes (Jang et al. 2020; Okbay et al. 2016) could be explained in part through genetic overlap between smoking and EA on the one hand and mental health and EA on the other. Overall, it seems that pleiotropy of genetic variants associated with EA play an important role in both smoking and mental health (Marees et al. 2020).

We further investigated the possibility that the different PGSs would show different profiles of G × E with environmental risk for smoking. If rGE between genetic effects on smoking and EA decreases the chance for detecting G × E, the PGS for smoking-without-EA (PGS_smok-noEA_) should be more sensitive to detect G × E. Alternatively, there was a possibility that people with a genetic susceptibility for a lower EA (PGS_EA_) would be more susceptible to environmental risk for smoking. Thus, we tested G × E of the PGS_smok-noEA_ and PGS_EA_ with neighborhood quality. None of the interactions survived correction for multiple testing, but there were suggestive effects that contributed some explained variance. Specifically, in *NTR,* a high PGS_allsmok_ was more likely to lead to smoking in lower-income neighborhoods, and a high PGS_EA_ was more likely to buffer against smoking in such neighborhoods. In *NEMESIS-2,* neighborhood quality had no main or interaction effects, so we used educational attainment as a proxy for SES. Here, a high PGS_smok-noEA_ was more likely to result in smoking for people with a higher educational attainment, whereas there were no differences between smokers and non-smokers in PGS_smok-noEA_ at low educational attainment. If neighborhood income and EA are regarded as aspects of the same underlying construct of SES, the G × E patterns in *NTR* and *NEMESIS-2* are incongruent (low SES amplified the PGS effects in *NTR* whereas high SES amplified PGS effects in *NEMESIS-2*). Furthermore, in *NTR* both smoking PGSs only had an effect on satisfaction with life at high neighborhood income, whereas no such G × E effects on mental health were observed in *NEMESIS-2*. These inconsistencies could suggest that G × E effects are specific to different aspects of the same environmental exposure (although alternative explanations, such as sample differences, are also possible). There were no clear patterns that could be discerned across samples and outcomes; the results did not clearly align with general models of differential susceptibility (Belsky and Pluess 2009) and did not show consistent differences between the type of PGS.

Important limitations of this study include the focus on smoking status rather than smoking quantity or nicotine dependence, which are more in-depth measures of smoking behavior and have been shown to be more heritable (Vink et al. 2005). However, given the need for statistical power we chose not to limit our analyses to sub samples of smokers, but rather used a general phenotype that was available for larger groups. Our use of the maximum sample size from the discovery sample (*UK Biobank*) and two independent target samples (*NTR* and *NEMESIS-2*) resulted in high power levels. We caution that the UK Biobank has been criticized based on its low representativeness and response rate which may affect (genetic) relationships between traits, although the extent of this seems small (Fry et al. 2017). While acknowledging this limitation, there is no more valuable resource in terms of sheer size, comprehensiveness, and accessibility than the UK Biobank. Although the *NEMESIS-2* sample size was limited, it included high-quality measures (especially of mental health), making it a valuable addition. The self-reported neighborhood quality measure did not predict smoking, which is not in line with previous literature. This could suggest that this measure does not reliably capture the neighborhood quality construct. Potentially, feelings toward the neighborhood constitute something inherently different than actual affluence (de Vries et al. 2020; Wen et al. 2006). Our use of different measures across the samples for SES (neighborhood-level income, self-reported neighborhood quality, and individual-level EA) and mental health (satisfaction with life and mental disorders) could be viewed as a limitation. It has certainly complicated the interpretation of the diverse G × E patterns that were observed. On the other hand, the use of different measures gives a more complete picture of the different aspects of the constructs of interest. It has alerted us to the presence of potential differences for specific (G × E) relationships tested. Another limitation includes the small effect sizes of the PGSs, which is a common limitation of the PGS method resulting from GWAS-identified genetic effects explaining only part of the trait. As a final limitation, our Genomic SEM model could not separate genetic effects on smoking that went via EA (i.e., were mediated by EA) from the total genetic effects for EA. Such ‘mediation’ variants might constitute a measure of vulnerability to EA circumstances, capturing the risk that a low EA would result in smoking behavior. A PGS based on such variants might be more likely to show interaction with environmental circumstances. Future research could aim to capture variants that increase *vulnerability to* an environmental exposure, rather than variants that simply increase the chance of being in such an environment.

The findings from this study have some important implications. We showed that, to some extent, genetic effects on EA could be subtracted from genetic effects on smoking, implying that besides overlap, there is also specificity in the genetic risk for EA and smoking. Focusing on specific genetic risk for smoking could improve precision of genetic prediction models and provide information on EA-independent etiological processes. This study has shown the feasibility and potential usefulness of dividing genetic predisposition in sub components, given that the components showed diverging patterns of overlap and their PGSs showed different main and interaction effects. This approach may be useful in other frameworks where it is important to tease apart pleiotropic and rGE effects, such as in Mendelian Randomization. The inconclusive G × E findings add to the mixed body of literature on G × E effects in substance use (Pasman et al. 2019). The fact that G × E effects did not reach significance and followed no clear pattern across different PGSs could be taken to suggest that G × E effects are small and specific to the individual relationships tested. The possibility that G × E effects are specific to the exact components that are in the PGS and in the environmental exposure opens up new lines for future research. Instead of reasoning from an overarching theoretical model (such as diathesis-stress or differential susceptibility) research could return to the drawing table and focus on testing interaction between specific genetic factors (e.g., ‘clean’ genetic risk factors, controlled for environmental covariates) and specific environmental factors (e.g., housing value). Furthermore, given the evidence for rGE, it seems hardly accurate to continue speaking of interaction with the environment, since environmental circumstances are not actually something separate from the individual and their genetic make-up. For example, subtracting EA resulted both in lower genetic correlations with SES-related (environmental) traits, as well as with intelligence (cognitive). Similarly, even the neighborhood where one lives is not strictly environmental but is under genetic influence (Laidley et al. 2019). Further layers of interplay can be added, and it is plausible that moderated rGE (e.g., where rGE effects differ per sub group; Wedow et al. 2018), or genetically mediated G × E effects exist (e.g., where vulnerability to G × E effects depend on some other genetic factor). Future research should be increasingly conscious about the meaning of statistical choices to model components as G, E, rGE, or G × E, and, preferably, test them concurrently.

Concluding, we show overlap and specificity in the genetic etiology of educational attainment and smoking. Gene-environment correlation plays an important role in the etiology of smoking. Although it has not provided definitive answers to our questions of G × E effects in substance use, we showed the feasibility of an approach based on modeling G × E using ‘partitioned’ genetic risk factors as a tool to investigate questions of overlap and interplay. Further refinements of this approach could contribute to disentangling the knot of genetic and environmental factors in the etiology of smoking and other complex traits, while providing further insight into where they overlap and interact.

## Supplementary Information

Below is the link to the electronic supplementary material.

Supplementary file1 (DOCX 29299 kb)

Supplementary file2 (XLSM 247 kb)

## References

[CR1] Abdellaoui A, Hugh-Jones D, Yengo L, Kemper KE, Nivard MG, Veul L (2019). Genetic correlates of social stratification in Great Britain. Nat Hum Behav.

[CR2] Arrindell WA, Heesink J, Feij JA (1999). The satisfaction with life scale (SWLS): appraisal with 1700 healthy young adults in The Netherlands. Person Individual Differ.

[CR3] Belsky J, Pluess M (2009). Beyond diathesis stress: Differential susceptibility to environmental influences. Psychol Bull.

[CR6] Boardman JD, Saint Onge JM, Haberstick BC, Timberlake DS, Hewitt JK (2008). Do schools moderate the genetic determinants of smoking?. Behav Genet.

[CR4] Boardman JD, Blalock CL, Pampel FC (2010). Trends in the genetic influences on smoking. J Health Soc Behav.

[CR5] Boardman JD, Blalock CL, Pampel FC, Hatemi PK, Heath AC, Eaves LJJD (2011). Population composition, public policy, and the genetics of smoking. Demography.

[CR7] Bommelé J, Willemsen M (2020) ijfers roken 2019: De laatste cijfers over roken, stoppen met roken en het gebruik van elektronische sigaretten. Trimbos

[CR8] Bulik-Sullivan BK, Loh P-R, Finucane HK, Ripke S, Yang J, Patterson N (2015). LD score regression distinguishes confounding from polygenicity in genome-wide association studies. Nat Genet.

[CR9] Buniello A, MacArthur JAL, Cerezo M, Harris LW, Hayhurst J, Malangone C (2019). The NHGRI-EBI GWAS catalog of published genome-wide association studies, targeted arrays and summary statistics 2019. Nucleic Acids Res.

[CR10] Cambron C, Kosterman R, Hawkins JD (2018). Neighborhood poverty increases risk for cigarette smoking from age 30 to 39. Ann Behav Med.

[CR11] Centraal Bureau voor de Statistiek (CBS) (2012) Kerncijfers postcodegebieden, 2008–2010. https://www.cbs.nl/nl-nl/maatwerk/2012/01/kerncijfers-postcodegebieden-2008-2010

[CR12] Cleveland HH, Wiebe RP, Rowe DC (2005). Sources of exposure to smoking and drinking friends among adolescents: a behavioral-genetic evaluation. J Genet Psychol.

[CR13] Cohen SS, Sonderman JS, Mumma MT, Signorello LB, Blot WJ (2011). Individual and neighborhood-level socioeconomic characteristics in relation to smoking prevalence among black and white adults in the Southeastern United States: a cross-sectional study. BMC Public Health.

[CR14] De Graaf R, Ten Have M, van Dorsselaer SJ (2010). The Netherlands mental health survey and incidence study-2 (NEMESIS-2): design and methods. Int J Methods Psychiatr Res.

[CR15] de Vries Y, ten Have M, de Graaf R, van Dorsselaer S, de Ruiter N, de Jonge P (2020). The relationship between mental disorders and actual and desired subjective social status. Epidemiology and Psychiatric Sciences.

[CR16] Demange PA, Malanchini M, Mallard TT, Biroli P, Cox SR, Grotzinger AD (2020). Investigating the genetic architecture of non-cognitive skills using GWAS-by-subtraction. Nat Genet.

[CR17] Dick DM, Viken R, Purcell S, Kaprio J, Pulkkinen L, Rose RJ (2007). Parental monitoring moderates the importance of genetic and environmental influences on adolescent smoking. J Abnorm Psychol.

[CR18] Diener E, Emmons RA, Larsen RJ, Griffin S (1985). The satisfaction with life scale. J Pers Assess.

[CR19] Dudbridge F, Fletcher O (2014). Gene–environment dependence creates spurious gene–environment interaction. Am J Hum Genet.

[CR20] Eichler EE, Flint J, Gibson G, Kong A, Leal SM, Moore JH, Nadeau JH (2010). Missing heritability and strategies for finding the underlying causes of complex disease. Nat Rev Genet.

[CR21] Fry A, Littlejohns TJ, Sudlow C, Doherty N, Adamska L, Sprosen T (2017). Comparison of sociodemographic and health-related characteristics of UK biobank participants with those of the general population. Am J Epidemiol.

[CR24] Gage SH, Smith GD, Ware JJ, Flint J, Munafo MR (2016). G = E: what GWAS can tell us about the environment. PLoS Genet.

[CR22] Gage SH, Bowden J, Davey Smith G, Munafò MR (2018). Investigating causality in associations between education and smoking: a two-sample Mendelian randomization study. Int J Epidemiol.

[CR23] Gage SH, Sallis HM, Lassi G, Wootton RE, Mokrysz C, Davey Smith G, Munafò MR (2020). Does smoking cause lower educational attainment and general cognitive ability? Triangulation of causal evidence using multiple study designs. Psychol Med.

[CR25] Grotzinger AD, Rhemtulla M, de Vlaming R, Ritchie SJ, Mallard TT, Hill WD (2018). Genomic SEM provides insights into the multivariate genetic architecture of complex traits. Nat Hum Behav.

[CR26] Harden KP, Hill JE, Turkheimer E, Emery RE (2008). Gene–environment correlation and interaction in peer effects on adolescent alcohol and tobacco use. Behav Genet.

[CR27] Hiscock R, Bauld L, Amos A, Fidler JA, Munafò MJ (2012). Socioeconomic status and smoking: a review. Ann N Y Acad Sci.

[CR28] International HapMap 3 Consortium et al (2010) Integrating common and rare genetic variation in diverse human populations. Nature 467(7311):52 10.1038/nature09298 PMC3173859 20811451

[CR29] Jang S-K, Saunders G, Liu M, Jiang Y, Liu DJ, Vrieze S (2020). Genetic correlation, pleiotropy, and causal associations between substance use and psychiatric disorder. Psychol Med.

[CR30] Karlsson Linnér R, Biroli P, Kong E, Meddens SFW, Wedow R, Fontana MA (2019). Genome-wide association analyses of risk tolerance and risky behaviors in over 1 million individuals identify hundreds of loci and shared genetic influences. Nat Genet.

[CR31] Karriker-Jaffe KJ (2013). Neighborhood socioeconomic status and substance use by U.S. adults. Drug Alcohol Depend.

[CR32] Karriker-Jaffe KJ, Liu H, Johnson RM (2016). Racial/ethnic differences in associations between neighborhood socioeconomic status, distress, and smoking among U.S. adults. J Ethn Subst Abuse.

[CR33] Keller MC (2014). Gene× environment interaction studies have not properly controlled for potential confounders: the problem and the (simple) solution. Biol Psychiatry.

[CR34] Kong A, Thorleifsson G, Frigge ML, Vilhjalmsson BJ, Young AI, Thorgeirsson TE (2018). The nature of nurture: effects of parental genotypes. Science.

[CR35] Kravitz-Wirtz N (2016). A discrete-time analysis of the effects of more prolonged exposure to neighborhood poverty on the risk of smoking initiation by age 25. Soc Sci Med.

[CR36] Laidley T, Vinneau J, Boardman J (2019). Individual and social genomic contributions to educational and neighborhood attainments: geography, selection, and stratification in the United States. Sociol Sci.

[CR37] Ligthart L, van Beijsterveldt CEM, Kevenaar ST, de Zeeuw E, van Bergen E, Bruins S (2019). The Netherlands Twin Register: longitudinal research based on twin and twin-family designs. Twin Res Hum Genet.

[CR38] Liu M, Jiang Y, Wedow R, Li Y, Brazel DM, Chen F (2019). Association studies of up to 1.2 million individuals yield new insights into the genetic etiology of tobacco and alcohol use. Nat Genet.

[CR39] Manolio TA, Collins FS, Cox NJ, Goldstein DB, Hindorff LA, Hunter DJ (2009). Finding the missing heritability of complex diseases. Nature.

[CR40] Marees AT, Smit DJA, Abdellaoui A, Nivard MG, van den Brink W, Denys D (2020). Genetic correlates of socio-economic status influence the pattern of shared heritability across mental health traits. Nat Hum Behav.

[CR41] Marioni RE, Davies G, Hayward C, Liewald D, Kerr SM, Campbell A (2014). Molecular genetic contributions to socioeconomic status and intelligence. Intelligence.

[CR42] Mathur C, Erickson DJ, Stigler MH, Forster JL, Finnegan JR (2013). Individual and neighborhood socioeconomic status effects on adolescent smoking: a multilevel cohort-sequential latent growth analysis. Am J Public Health.

[CR43] McCaffery JM, Papandonatos GD, Lyons MJ, Koenen KC, Tsuang MT, Niaura RJ (2008). Educational attainment, smoking initiation and lifetime nicotine dependence among male Vietnam-era twins. Psychol Med.

[CR44] Meyers JL, Cerda M, Galea S, Keyes KM, Aiello AE, Uddin M (2013). Interaction between polygenic risk for cigarette use and environmental exposures in the Detroit Neighborhood Health Study. Transl Psychiatry.

[CR46] Miles R (2006). Neighborhood disorder and smoking: findings of a European urban survey. Soc Sci Med.

[CR47] Okbay A, Baselmans BML, De Neve J-E, Turley P, Nivard MG, Fontana MA (2016). Genetic variants associated with subjective well-being, depressive symptoms, and neuroticism identified through genome-wide analyses. Nat Genet.

[CR48] Pasman JA, Smit K, Volleberg W, Nolte IM, Hartman C, Abdellaoui A, Verweij KJH, Maciejewski D, Vink JM (2021). Interplay between genetic risk and the parent environment in adolescence and substance use in young adulthood: a TRAILS study. Dev Psychopathol.

[CR49] Pasman JA, Verweij KJ, Abdellaoui A, Hottenga JJ, Fedko IO, Willemsen G (2020). Substance use: interplay between polygenic risk and neighborhood environment. Drug Alcohol Depend.

[CR50] Pasman JA, Verweij KJ, Vink JM (2019). Systematic review of polygenic gene–environment interaction in tobacco, alcohol, and cannabis use. Behav Genet.

[CR51] Plomin R, DeFries JC, Loehlin JC (1977). Genotype–environment interaction and correlation in the analysis of human behavior. Psychol Bull.

[CR52] Purcell S, Neale B, Todd-Brown K, Thomas L, Ferreira MAR, Bender D (2007). PLINK: a tool set for whole-genome association and population-based linkage analyses. Am J Hum Genet.

[CR53] Rathouz PJ, Van Hulle CA, Rodgers JL, Waldman ID, Lahey BB (2008). Specification, testing, and interpretation of gene-by-measured-environment interaction models in the presence of gene–environment correlation. Behav Genet.

[CR54] Robinson MR, Kleinman A, Graff M, Vinkhuyzen AAE, Couper D, Miller MB (2017). Genetic evidence of assortative mating in humans. Nat Hum Behav.

[CR55] Schmitz L, Conley D (2016). The long-term consequences of Vietnam-era conscription and genotype on smoking behavior and health. Behav Genet.

[CR56] Shen H, Feldman MW (2020). Genetic nurturing, missing heritability, and causal analysis in genetic statistics. Proc Natl Acad Sci USA.

[CR57] Sullivan PF, Kendler KS (1999). The genetic epidemiology of smoking. Nicotine Tobacco Res.

[CR58] The Tobacco and Genetics Consortium (2010). Genome-wide meta-analyses identify multiple loci associated with smoking behavior. Nat Genet.

[CR59] Timberlake DS, Rhee SH, Haberstick BC, Hopfer C, Ehringer M, Lessem JM (2006). The moderating effects of religiosity on the genetic and environmental determinants of smoking initiation. Nicotine Tobacco Res.

[CR60] Treur JL, Verweij KJ, Abdellaoui A, Fedko IO, de Zeeuw EL, Ehli EA (2018). Testing familial transmission of smoking with two different research designs. Nicotine Tobacco Res.

[CR61] UNESCO Institute for Statistics (2011) International Standard Classification of Education: ISCED 2011, Montreal, Canada

[CR62] Verhulst B, Hatemi PK (2013). Gene–environment interplay in twin models. Polit Anal.

[CR63] Vink JM, Boomsma DI (2011). Interplay between heritability of smoking and environmental conditions? A comparison of two birth cohorts. BMC Public Health.

[CR64] Vink JM, Willemsen G, Boomsma DI (2005). Heritability of smoking initiation and nicotine dependence. Behav Genet.

[CR65] Vinkhuyzen AAE, Van Der Sluis S, De Geus EJC, Boomsma DI, Posthuma D (2010). Genetic influences on ‘environmental’ factors. Genes Brain Behav.

[CR66] Wang J, Li MD (2010). Common and unique biological pathways associated with smoking initiation/progression, nicotine dependence, and smoking cessation. Neuropsychopharmacology.

[CR67] Watanabe K, Taskesen E, van Bochoven A, Posthuma D (2017) Functional mapping and annotation of genetic associations with FUMA. Nat Commun 8(1):1826 10.1038/s41467-017-01261-5 PMC5705698 29184056

[CR68] Wedow R, Zacher M, Huibregtse BM, Mullan Harris K, Domingue BW, Boardman JD (2018). Education, smoking, and cohort change: forwarding a multidimensional theory of the environmental moderation of genetic effects. Am Sociol Rev.

[CR69] Wen M, Hawkley LC, Cacioppo JT (2006). Objective and perceived neighborhood environment, individual SES and psychosocial factors, and self-rated health: an analysis of older adults in Cook County, Illinois. Soc Sci Med.

[CR70] Wills AG, Carey G (2013). Adolescent peer choice and cigarette smoking: evidence of active gene–environment correlation?. Twin Res Hum Genet.

[CR71] World Health Organization (2019). WHO report on the global tobacco epidemic 2019: offer help to quit tobacco use.

[CR72] Yang J, Lee SH, Goddard ME, Visscher PM (2011). GCTA: a tool for genome-wide complex trait analysis. Am J Hum Genet.

