# Supplementary material for: Genetic Risk for Smoking: Disentangling Interplay Between Genes and Socioeconomic Status
Source: Behav Genet. 2021 Dec 2;52(2):92–107. doi: 10.1007/s10519-021-10094-4 (PMC8860781; doi:10.1007/s10519-021-10094-4)
Supplement: Supplementary file 1 — Supplementary file1 (DOCX 29299 kb) [file 10519_2021_10094_MOESM1_ESM.docx]

**Genetic risk for smoking: disentangling gene environment interplay with socioeconomic status**

**Supplemental figures**

Contents

[Figure S1. Manhattan-plot for the smoking GWAS in UK-Biobank (N=454,195). 2](#_Toc74313267)

[Figure S2. Manhattan-plot for the educational attainment GWAS in UK-Biobank (N=451,800). . 2](#_Toc74313268)

[Figure S3. Gene-based Manhattan-plot for the smoking-without-EA GWAS 3](#_Toc74313269)

**
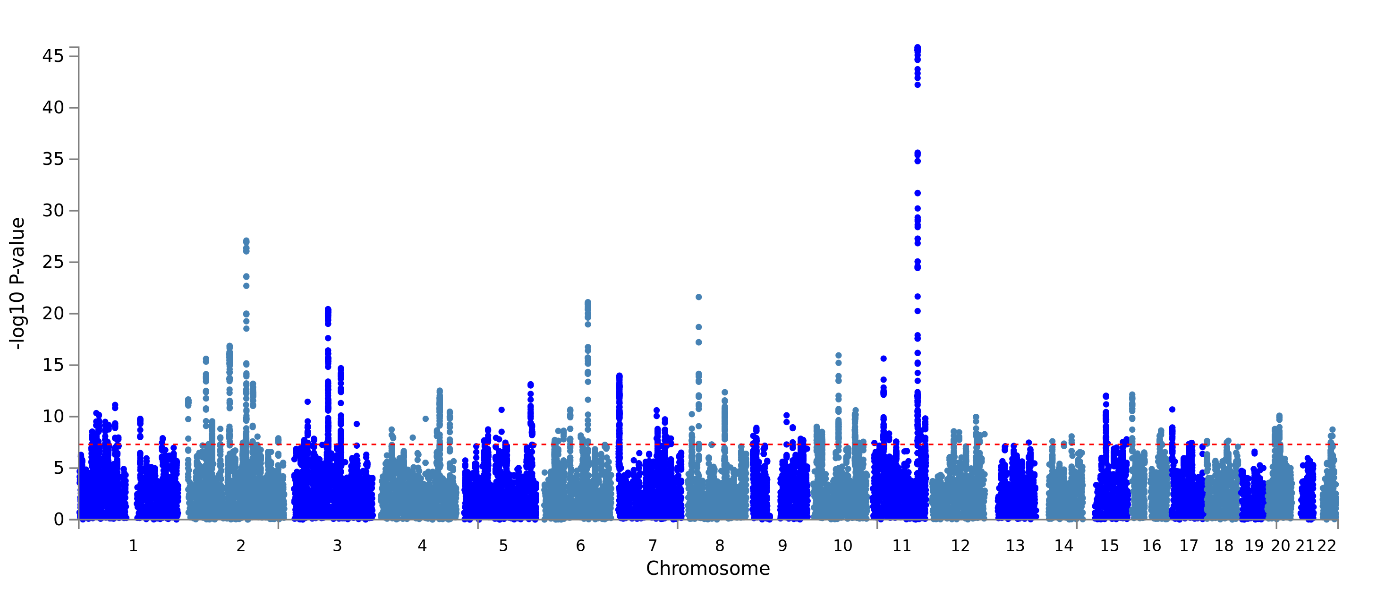
**

# Figure S1. Manhattan-plot for the smoking GWAS in UK-Biobank (N=454,195). SNPs with a log-10 p-value smaller than 5E-08 (i.e., exceeding the red line) are genome-wide significant hits.


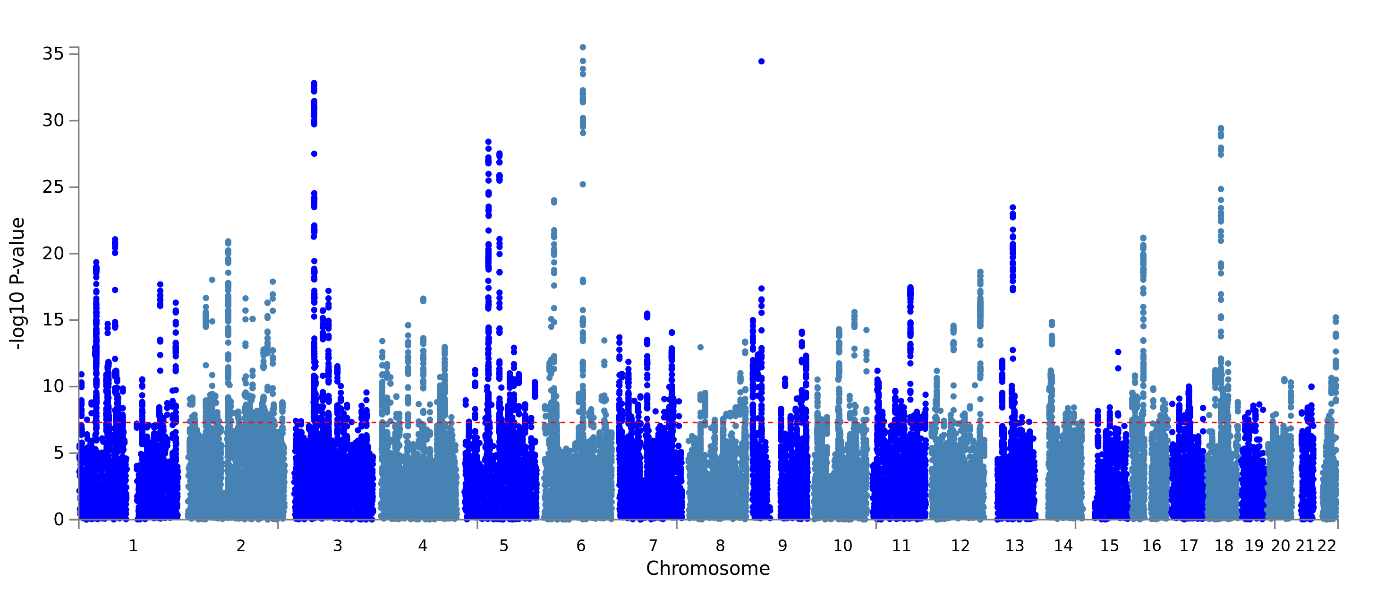


# Figure S2. Manhattan-plot for the educational attainment GWAS in UK-Biobank (N=451,800). SNPs with a log-10 p-value smaller than 5E-08 (i.e., exceeding the red line) are genome-wide significant hits.

Figure S3. Gene-based Manhattan-plot for the smoking-without-EA GWAS, defined in Genomic SEM by subtracting the genetic signal in the smoking GWAS that overlapped with the EA GWAS**.** Genes with a p-value smaller than 3.201e-6 (i.e., exceeding the red line) are genome-wide significant genes. This p-value was corrected for 15,620 independent tests (the number of protein coding genes).
